# Targeting Trop2 in solid tumors: a look into structures and novel epitopes

**DOI:** 10.3389/fimmu.2023.1332489

**Published:** 2023-12-20

**Authors:** Xinlin Liu, Jiyixuan Li, Junwen Deng, Jianan Zhao, Gaoxiang Zhao, Tingting Zhang, Hongfei Jiang, Bing Liang, Dongming Xing, Jie Wang

**Affiliations:** ^1^The Affiliated Hospital of Qingdao University, Qingdao University, Qingdao, China; ^2^Qingdao Cancer Institute, Qingdao, China; ^3^School of Life Sciences, Tsinghua University, Beijing, China

**Keywords:** Trop2, structure, epitopes, tumor, targeted therapy

## Abstract

Trophoblast cell surface antigen 2 (Trop2) exhibits limited expression in normal tissues but is over-expressed across various solid tumors. The effectiveness of anti-Trop2 antibody-drug conjugate (ADC) in managing breast cancer validates Trop2 as a promising therapeutic target for cancer treatment. However, excessive toxicity and a low response rate of ADCs pose ongoing challenges. Safer and more effective strategies should be developed for Trop2-positive cancers. The dynamic structural attributes and the oligomeric assembly of Trop2 present formidable obstacles to the progression of innovative targeted therapeutics. In this review, we summarize recent advancements in understanding Trop2’s structure and provide an overview of the epitope characteristics of Trop2-targeted agents. Furthermore, we discuss the correlation between anti-Trop2 agents’ epitopes and their respective functions, particularly emphasizing their efficacy and specificity in targeted therapies.

## 1 Introduction

Trop2, encoded by the *TACSTD2* gene, is a type I surface glycoprotein (1, 2). It is also referred to as pancreatic carcinoma marker protein GA733–1/GA733, gastrointestinal tumor-associated antigen GA7331, epithelial glycoprotein-1 (EGP-1), membrane component chromosome 1 surface marker 1 (M1S1), CAA1, and TTD2 (3). Trop2 plays an essential role in the development of embryonic organs and shows restricted expression levels in normal tissues (4). Conversely, overexpressed Trop2 has been observed in various tumor types, including breast cancer (BC), non-small-cell lung cancer (NSCLC), oral squamous cell carcinoma (OSCC), salivary gland carcinomas (SGC), thyroid cancer (TC), gastric cancer (GC), pancreatic cancer (PC), gallbladder cancer, colorectal cancer (CRC), prostate cancer, ovarian cancer, cervical cancer and urothelial cancer (UC). It’s found that overexpression of Trop2 correlates with tumor invasion, metastasis, and poor prognosis (5–10). Numerous binding partners for Trop2 have been identified, including insulin-like growth factor 1 (IGF-1), claudins 1 and 7, cyclin D1, tumor necrosis factor α-converting enzyme (TACE), and protein kinase C (PKC). The anti-Trop2 ADC (Trodelvy ™, sacituzumab govitecan, SG), which has been approved in metastatic BC and metastatic UC, proves Trop2 to be a valid therapeutic target in tumor treatment (11–13). The positive results encourage the clinical development of innovative Trop2-targeted ADC (14, 15). However, the challenge remains due to their poor response and unfavorable risk-benefit profiles. Hence, there is an urgent need to develop safer and more effective strategies based on the biological and structural characteristics of Trop2. This review encapsulates recent advancements in Trop2 structural research, critically supporting further developments and facilitating future endeavors in the design of targeted therapeutics. Next, we proceed to explore the characteristics of epitopes for Trop2-targeted agents, highlighting the relationship between binding epitopes and their efficacy.

## 2 Structure of Trop2

**Figure 1:**
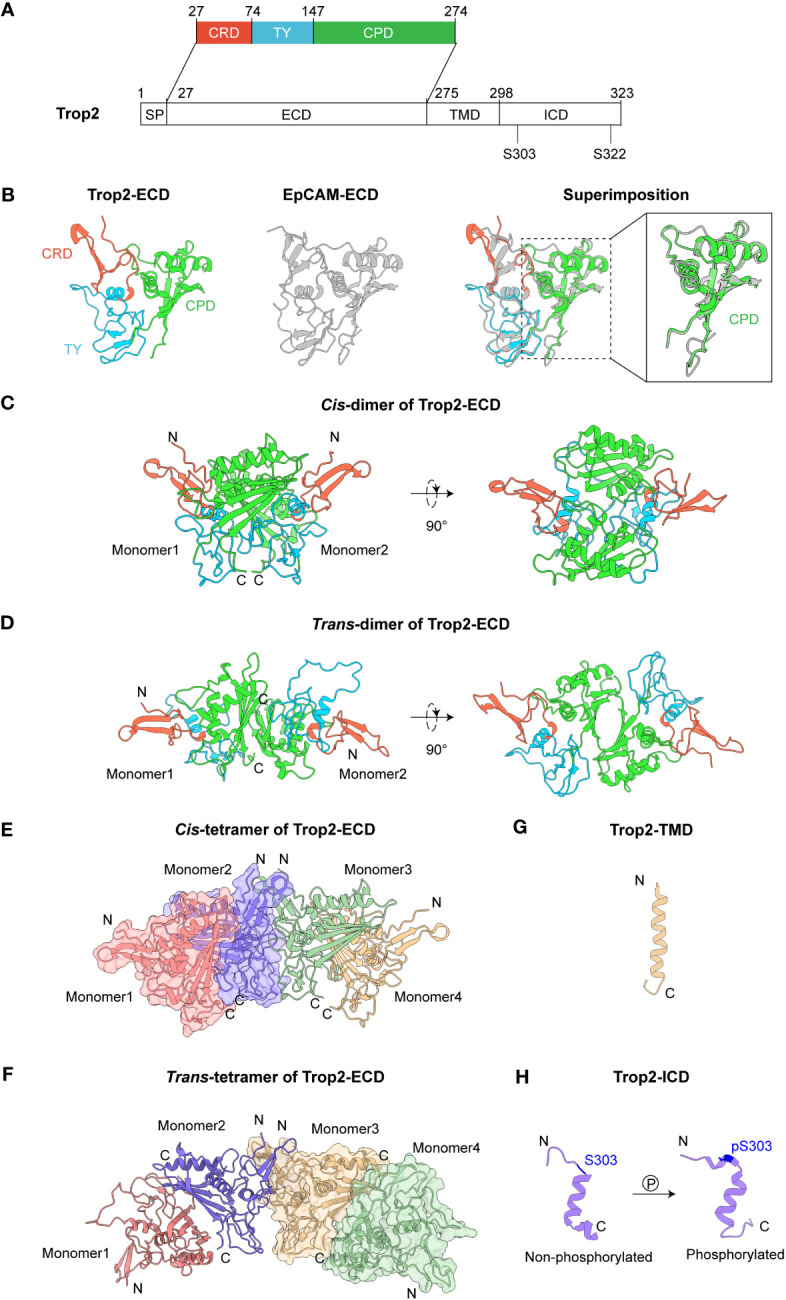
Structures of Trop2. **(A)** Domain architecture of Trop2. **(B)** Structures of Trop2-ECD (PDB 7PEE) and EpCAM-ECD (PDB 4MZV). Superimposition of Trop2-ECD and EpCAM-ECD illustrates their similar conformations. The enlarged part shows high structural similarity between Trop2-ECD-CPD and EpCAM-ECD-CPD, with RMSD at 0.879 (Å) **(C)** *Cis-*dimer of Trop2-ECD (PDB 7E5N). **(D)** *Trans-*dimer of Trop2-ECD (PDB 7E5M). **(E)** *Cis-*tetramer of Trop2-ECD (PDB 7E5N). **(F)** *Trans-*tetramer of Trop2-ECD. This tetramer formation could be obtained via a simple symmetrical operation on *trans-*dimer. **(G)** Structure of Trop2-TMD from NMR and MD (PDB 2MAE). **(H)** Structures of non-phosphorylated (PDB 2MVL) and phosphorylated forms (PDB 2MVK) of Trop2-ICD.

The Trop2 protein, with a full length of 323 amino acids (AA), is composed of four domains, including a signal peptide (SP), an extracellular domain (ECD), a single transmembrane helix (TMD) and a cytoplasmic tail (ICD) (**Figure 1A**). These domains jointly enable the complex functionalities of Trop2, encompassing oligomerization, cell-cell communication and downstream signaling regulation. We subsequently summarize the recent advances in Trop2 structural studies, which will contribute to a deeper comprehension of the mechanisms underlying its oncogenic behaviors.

### 2.1 Extracellular domain (ECD)

As the largest part of the molecule, Trop2-ECD (H27-T274) comprises three subdomains: a cysteine-rich domain (CRD), a thyroglobulin type-1 domain (TY), and a cysteine-poor domain (CPD). Trop2-ECD undergoes post-translational modification at four N-glycosylation sites (N33Q, N120Q, N168Q, and N208Q). Earlier studies employed conventional biochemical methods to investigate the structure and functions of Trop2, but these indirect approaches did not reveal the intricate molecular mechanisms underlying Trop2 biology. Recent advances in structural biology have enabled high-resolution structures of Trop2-ECD, providing structural insights into the oligomeric assembly of Trop2 (16, 17). Trop2-ECD shares 47.6% sequence identity with the ECD of epithelial cell adhesion molecule (EpCAM). Crystal analysis revealed that Trop2-ECD also presents a similar conformation to EpCAM-ECD with a root-mean-square deviation (RMSD) of 0.780 Å (**Figure 1B**). CRD’s stability is achieved via the formation of three disulfide bonds (C34-C53, C36-C66, and C44-C55), and another three disulfide bonds (C73-C108, C119-C125, C127-C145) provide stability to the TY domain. Two α-helices (α2, α3) and a βC sheet comprising four β ribbons (βC1-4) are observed in the C-termini of CPD. Notably, the extended loop between α3 and βC4 of Trop2-CPD demonstrates a conformation that is distinct from EpCAM (18, 19).

Utilizing two protein expression systems, Sun et al. demonstrated that Trop2-ECD can self-assemble to *cis-* or *trans-*dimers, and even tetramers (17). At the interface of *cis*-dimer, a complicated hydrogen bond interaction network forms between TY-loop of one monomer and the βC sheet of the other (**Figure 1C**). Contrarily, in the case of Trop2-ECD *trans-*dimer, the interaction is primarily mediated by the βC sheet of two monomers’ CPD, which covers a comparatively smaller surface area than *cis*-dimer (**Figure 1D**). The stability of *trans-*dimeric formation is facilitated by three hydrogen bonds interaction (R206-H152’, D157-Y259’, and D101-E197’). Superimposition of *cis-* and *trans-*dimers of Trop2-ECD reveals a pronounced overlapped interacting surface, indicating that these two formations could be mutually exclusive. Interestingly, the tetrameric assembly of two *cis-* or *trans-*dimers depends on the N-termini of CRD of Trop2-ECD, which has no spatial steric hindrance with the interface of dimerization (**Figures 1E, F**). This consequently provides a potential structural model to explain the large clustering of Trop2 on the tumor cell surface (5, 6, 20).

Tumor-specific proteolytic cleavage of Trop2, induced by ADAM10 at R87-T88 of TY domain, demonstrated an activator switch for tumor growth and metastasis (21, 22). These cleavage sites are accessible in both *cis-* and *trans-*dimerization of Trop2-ECD. Additionally, Trerotola et al. reported that this ADAM10-mediated cleavage might trigger a profound rearrangement of Trop2-ECD (22). Sun et al. used a truncated Trop2-△Q31-R88 protein to assess the cross-linking level of Trop2-ECD. The results showed that Trop2-ECD retains the ability of dimeric assembly, in which the free C108 of TY possibly forms disulfide bonds to stabilize the dimer post Q31-R88 depletion. The detailed assembly pattern of Trop2 following tumor-specific proteolytic cleavage needs further investigation. Additionally, another potential cleavage site between A193-V194 mediated by TACE/ADAM17 also remains accessible in the dimeric formations of Trop2-ECD (16, 23). Recently, Guerra et al. demonstrated that Trop-2, Na^+^/K^+^ ATPase, CD9, PKCα, and cofilin assemble a membrane signaling super-complex, driving CRC growth and invasion (24). The formation of the complex can initiate proteolytic cleavage of E-cadherin, remodel the β-actin cytoskeleton, and activate the downstream signaling pathways of Akt and ERK. Furthermore, the high expression level of Trop2-super-complex determines a poor disease outcome in CRC patients. The researcher speculated the assembly of this super-complex might be triggered by clustering and proteolytic cleavage of Trop2-ECD or other yet unidentified mechanisms.

### 2.2 Transmembrane domain (TMD) and intracellular domain (ICD)

Trop2-ECD is anchored to the membrane via a transmembrane helix domain (TMD), which links to the intracellular domain (ICD). However, the notable conformational flexibility poses difficulty in resolving the high-resolution structure of Trop2-TMD within the full-length Trop2. Pavšič et al. used the molecular dynamics (MD) approach in examining the spatial structural characteristics of Trop2-TMD as it is embedded in a lipid bilayer (25) (**Figure 1G**). The MD simulation of two canonical α-helices illustrated that Trop2-TM tends to form a transmembrane dimer, with a “VVVVV” motif (V282-V286) constituting the predominant interaction. This dimeric propensity of Trop2-TMD could bring the ICD of two Trop2 molecules closer, potentially facilitating the downstream Trop2 signaling activation (7).

Trop2-ICD, a cytoplasmic tail consisting of 26 amino acids (T298-L323), serves as core transmitting signaling. It contains a highly conserved phosphatidylinositol-4,5-bisphosphate (PIP2) binding sequence and two serine phosphorylation sites (S303 and S322), which can regulate the cell cycle progression and cell motility (26, 27). The intramembrane hydrolysis mediated by ADAM10 or TACE can induce the release of Trop2-ICD and then promote their accumulation in the nucleus, resulting in the upregulation of cyclin D1 and proto-oncogene c-myc (23). Nuclear magnetic resonance (NMR) structures of Trop2-ICD illustrated that the central amino acids (K305-L314) can form an α-helix. Through the comparison of non-phosphorylated and phosphorylated forms of Trop2-ICD, Pavšič et al. identified that phosphorylation (S303) of Trop2-ICD mediates salt bridge reshuffling, leading to pronounced conformational changes including ordering of the C-terminal tail (25) (**Figure 1H**). Moreover, this phosphorylation-triggered reorganization of Trop2-ICD could induce the formation of a hydrophobic cluster (I311, E316, and S322), leading to their association with membrane and providing accessibility for Trop2’s binding partners.

## 3 Functions and binding epitopes of Trop2-targeted agents

Despite Trop2’s identification as early as 1995, only in recent years did breakthroughs occur in Trop2-targeted therapies (13, 28–30). Various anti-Trop2 agents, including ADC, monoclonal antibody (mAb), bispecific antibody (biAb), fusion protein, and chimeric antigen receptor (CAR) T-cell therapy, have been developed to improve the clinical outcomes of patients with Trop2-positive tumors (31, 32). The large ECD makes it the ideal therapeutic target for anti-Trop2 agents that are engineered to interfere with oncogenic functions. Therefore, the primary direction of current Trop2-targeted therapeutics is agents based on antibodies (**Table 1**). Below, the epitopes of Trop2-targeted antibody-based agents are described and the efficacy and limitations of each strategy are discussed.

**Table 1:** The Trop2-targeted antibodies in development.

Agent	Institution/Company	Classification	Binding Epitope	Phase	Reference
**RS7**	Immunomedics, Glead Science	Antibody	RCPD (Q237-Q252)	Preclinical	(17)
**PrE11**	Kyowa Hakko Kirin	Antibody	CRD (C34-K72)	Preclinical	(33, 34)
**Trop2-IgG**	Nanjing Medical University	Antibody	–	Preclinical	(35)
**AR47A6.4.2**	ARIUS Research	Antibody	CPD (L179-H187 and Q252-Y260)	Preclinical	(36)
**77220, MOv16, MM0588-49D6, YY-01, 162-46.2, T16, E1**	Kyowa Hakko Kirin	Antibody	CPD (D146-R178)	Preclinical	(22, 33, 37)
**7E6**	Michigan State University	Antibody	CPD (D171, R178, and G241-P250)	Preclinical	(38)
**TrMab-6**	Tohoku University	Antibody	–	Preclinical	(39)
**TrMab-29**	Tohoku University	Antibody	–	Preclinical	(40)
**K5-70**	Chiome Bioscience	Antibody	CRD (V43-D65)	Preclinical	–
**hIMB1636**	Chinese Academy of Medical Sciences & Peking Union Medical College	Antibody	–	Preclinical	(41)
**2EF**	University of Messina	Antibody	CRD or TY	Preclinical	(42)
**2G10**	University of Messina	Antibody	CPD	Preclinical	(43)
**Trop2-Fab**	Nanjing Medical University	Fab	–	Preclinical	(44)
**(E1)-3s**	Immunomedics	Bispecific antibody (Trop2/CD3)	RCPD (Q237-Q252)	Preclinical	(45)
**F7AK3**	Huazhong University of Science and Technology Tongji Medical College	Bispecific antibody (Trop2/CD3)	–	Preclinical	(46)
**Anti-Trop2/CD3 bispecific antibody**	Sunshine Guojian Pharmaceutical	Bispecific antibody (Trop2/CD3)	CRD, C34-K72	Preclinical	(47)
**TF12**	Radboud University Medical Center	Bispecific antibody (Trop2/HSG)	–	Preclinical	(48)

### 3.1 Targeting CRD and TY domains of Trop2

Trop2-CRD is located on the N-terminal region of Trop2-ECD and is responsible for the tetrameric assembly of dimers. Thus, targeting epitopes of CRD possesses the capacity to interfere with the dynamic behavior of Trop2, leading to an interruption in Trop2 activation. In 2015, Ikeda et al. obtained an anti-Trop2 antibody, named Pr1E11, via an adenovirus-based antibody screening (33). The analysis of domain-deletion constructions of Trop2-ECD revealed that Pr1E11 recognizes a unique epitope at the CRD region. Although Pr1E11 demonstrated a weak internalization activity and had no inhibitory effects on tumor cell proliferation *in vitro*, it exhibited considerable antitumor activity due to better cell surface retention and ADCC *in vivo* (34). Pr1E11 was subsequently used to engineer a Trop2/CD3 biAb, comprising of the anti-CD3 mAb scFv inserting into Pr1E11 (47). This bispecific construct demonstrated potent tumor-killing activity and resulted in reduced induction of Th1 cytokines. These results indicate that Pr1E11, when used as a naked mAb, is insufficient to obstruct the growth of Trop2-positive tumor cells and its functionality depends on immune effects. Further study is required to determine whether the CRD-binding of Pr1E11 interferes with the oligomeric state of Trop2 on the tumor cell surface, a critical factor for Trop2-mediated tumor progression. K5-70, an anti-Trop2 mAb developed by Chiome Bioscience (CN107236043B), demonstrated potent antitumor efficacy *in vivo* in various tumor models, including SW480, DU-145, and PK-59. Furthermore, it exhibits effective suppression of a recurrent tumor model previously treated with irinotecan hydrochloride. Chemically linked peptides on scaffolds technology (CLIPS) indicated that K5-70 mainly targets the polypeptide (V43-D65) within Trop2-CRD. Structural analysis showed that the binding epitope of K5-70 is involved in the tetramerization interface mediated by the N-terminal CRD, suggesting its promising suppressive effects might be facilitated by the disruption of Trop2 clustering on the tumor cell surface.

The TY domain plays a critical role in forming the stable Trop2-ECD *cis-*dimer and contains conserved tumor-specific proteolytic cleavage sites (R87-T88). Targeting the epitopes at the TY domain seems a promising antagonist strategy to hinder the ordered assembly of Trop2, potentially suppressing Trop2-associated interactions and activation. Recently, Guerra et al. reported a novel anti-Trop2 mAb 2EF that targets an N-terminal epitope of Trop2-ECD (42). Analysis of recombinant Trop2 deletion mutants revealed that 2EF recognizes either CRD or TY domain. 2EF demonstrated the capability to bind to Trop2 at cell-cell junctions in MCF-7 breast cancer cells, and at deeply located sites in prostate cancer previously inaccessible to other anti-Trop-2 antibodies (T16). It showed inhibitory effects on CRC cell growth *in vitro*, particularly displaying increased activity at high cell densities. The antitumor activity of 2EF was observed in multiple tumor models *in vivo*, including SKOV3, COLO205, HT29, HCT116, and DU145. Considering the non-overlapping recognition of 2EF and 2G10 (a Trop2-targeted mAb selectively binding cleaved Trop2), the researcher proposed that their combination could exert synergistic antitumor efficacy. Predictably, 2EF significantly enhanced the *in vivo* antitumor effects of 2G10 in Trop2-positive tumor models.

### 3.2 Targeting Trop2-CPD

The CPD region, a stem part of Trop2-ECD, provides a substantial accessible surface for the binding of anti-Trop2 agents. The region plays a role in both *cis*-dimerization and *trans*-dimerization of Trop2-ECD. Most existing literature on Trop2-targeted drugs reports recognition of sites within the CPD region, suggesting the presence of multiple immunodominant epitopes in this region (49). RS7 mAb, the antibody component of approved anti-Trop2 ADC SG, was characterized by Stein and colleagues in 1990 (50, 51). While it shows potent internalization activity, this mAb has no therapeutic activity in its unconjugated form (52, 53). The analysis of domain-substituted Trop2 mutants demonstrated that RS7 recognizes a linear epitope (Q237-Q252, also referred to as RCPD) within the CPD region. The exposed loop is distant from the interfaces of *cis*- and *trans*-dimerization, indicating RS7 is unable to disrupt the self-assembly of Trop2-ECD (17). This could account for why RS7 alone is ineffective in suppressing the growth of Trop2-positive tumors *in vivo*. Thus, the tumor-killing activity of existing RS7-based ADCs mainly relies on the toxicity of payloads following specific binding to Trop2-expressed tumor cells (54). Additionally, humanized RS7 has been used to construct biAbs, fusion proteins, and CAR-T owing to its high affinity towards Trop2 (45, 55). It remains unclear, though, whether these RS7-based anti-Trop2 therapeutics interfere with the biological functions of Trop2. Much like RS7, another batch of mAbs, which includes 162-46.2, T16, MOv-16 (37), 77220, MM0588-49D6, YY-01 (33), and E1 (22), have limited therapeutic effects, partly because their binding epitopes (D146-R178) are far from the key interaction surface of Trop2 dimeric association.

AR47A6.4.2, produced through ARIUS’ FunctionFIRST™ platform, exhibited a significant tumor growth inhibition in human models of breast (90%, p<0.00001), colon (60%, p<0.001), and prostate (60.9%, p<0.001) cancer (36). This promising antitumor activity is attributable to at least two mechanisms of action (MOAs): complement-dependent cytotoxicity (CDC) and downregulation of MAPK signaling pathway. Epitope mapping experiments confirmed that AR47A6.4.2 recognized two liner epitopes (L179-H187 and Q252-Y260) within Trop2-CPD. Notably, the Q252-Y260 epitope overlaps with the interface of both *cis*-dimer and *trans*-dimer, suggesting that AR47A6.4.2 might suppress tumor growth by the blockade of dimeric formations of Trop2.

7E6, a mouse mAb primarily binding to the C-terminal of CPD, showed significant inhibitory activity in the A431 xenograft model (US8871908B2). A. Kowalsky and colleagues constructed single site saturation mutagenesis (SSM) libraries to determine that D171, R178, and G241-P250 contribute to the 7E6-Trop2 interaction (38). Despite its antitumor effects *in vivo*, 7E6 did not inhibit tumor cell proliferation *in vitro* but did mediate reduced migration. Confocal images revealed that the nuclear expression and localization of Trop2-ICD were retained in 7E6-treated cells. These findings suggest that the inhibitory activity of 7E6 could be facilitated by blocking the agonist binding or interfering with the downstream signaling cascade.

Previous studies indicated that the immunodominant epitopes appear to be equally accessible in both tumors and normal cells (5, 37, 56). Such poor tumor-specificity of anti-Trop2 agents might result in the exposure of normal tissues, leading to potentially unmanageable toxicity (57). Given that tumor-specific cleavage induced by ADAM10 triggers the conformational rearrangement of Trop2-ECD and might expose previously inaccessible sites, Alberti and colleagues recently used deletion mutagenesis without immunodominant epitopes to generate a cancer-specific CPD-targeted mAb 2G10 (58). It shows a higher affinity (Kd < 10^−12^ mol/L) towards cleaved/activated Trop2 in tumor cells compared to uncleaved/wtTrop2 in normal cells (43). Humanized 2G10 (Hu2G10) demonstrated *in vivo* inhibition of various tumor types, including breast, colon, ovary, and prostate cancers. No systemic toxicity was observed following Hu2G10 treatment. The ACD setting of Hu2G10, namely LCB84, demonstrated potent effectiveness against multiple Trop2-positive cell-line derived xenograft (CDX) models, including triple-negative breast cancer (TNBC), pancreatic ductal adenocarcinoma (PDAC), GC and NSCLC (59). This novel 2G10-based strategy, which recognizes tumor-specific Trop2, possesses the potential to improve the clinical outcome of next-generation Trop2-targeted therapies.

## 4 Conclusions and perspectives

The considerable advancements in antibody-based anti-Trop2 therapies have reshaped the treatment landscape for Trop2-positive solid tumors. Accumulated expertise on the mechanism of Trop2-mediated oncogenic activity, together with an enriched understanding of structural biology, has driven the progress of next-generation therapies. However, it remains unresolved whether patients with Trop2-positive cancers would benefit from alternative agents targeting other available epitopes, specifically the safe and tumor-specific sites. It’s worth noting that a significant number of anti-Trop2 candidate drugs still have unclear epitope information (39, 40, 46, 48, 60–63). Therefore, the pursuit to discover more potent Trop2-targeted therapeutics based on novel druggable epitopes and to further investigate the relationship between epitopes and MOAs of anti-Trop2 agents continues to be a crucial avenue for future research. The second aspect to consider for future research is to elucidate the full-length structure of the Trop2. Regrettably, currently, all available high-resolution structural models of Trop2 are based on uncoupling domains, resulting in a lack of a comprehensive view of the dynamics involving intact receptor activation and antigen-antibody interactions. Despite the identification of numerous anti-Trop2 agents and Trop2-binding partners, their detailed interaction mechanisms remain ambiguous, partly due to the lack of structures of Trop2-containing complexes. This incomplete structural information constrains the structure-guided design of optimum inhibitors, which could provide a more comprehensive disruption of Trop2-centered signaling pathways.

In conclusion, we must maintain our unceasing dedication to deepening our understanding of Trop2’s structural biology. Unraveling the complex mechanisms underlying the varied therapeutic functions induced by distinct epitope binding would aid in the rational design of Trop2-targeted strategies and increase opportunities to maximize clinical benefits for Trop2-positive tumor patients.

## Author contributions

XL: Funding acquisition, Resources, Writing – original draft, Writing – review & editing. JL: Writing – original draft. JD: Writing – original draft. JZ: Writing – review & editing. GZ: Writing – review & editing. TZ: Writing – review & editing. HJ: Writing – review & editing. BL: Writing – review & editing. DX: Writing – review & editing. JW: Writing – review & editing, Conceptualization.
